# Case report: Epilepsy during the use of recombinant human growth hormone: a report on two cases and a literature review

**DOI:** 10.3389/fphar.2024.1458487

**Published:** 2024-09-12

**Authors:** Yuan Zhou, Ruofan Jia, Zhuangjian Xu, Yaping Ma

**Affiliations:** ^1^ Department of Pediatrics, Affiliated Hospital of Jiangnan University, Wuxi, Jiangsu, China; ^2^ Wuxi School of Medicine, Jiangnan University, Wuxi, Jiangsu, China

**Keywords:** recombinant human growth hormone, epilepsy, boy, short stature, gonadotrophin-releasing hormone analog

## Abstract

**Background:**

Epilepsy during recombinant human growth hormone (rhGH) therapy is rare in children. The potential association between rhGH treatment and epilepsy remains unclear.

**Methods:**

We retrospectively analyzed the clinical data of two Chinese boys who experienced epilepsy during the use of rhGH and reviewed the relevant literature.

**Results:**

Case 1, an 8-year and 2-month-old boy, was diagnosed with short stature, malnutrition, and congenital hypothyroidism. He was on levothyroxine sodium tablets for a long time. Recurrent febrile convulsions were present at 6–7 years. Electroencephalogram and magnetic resonance imaging (MRI) showed no abnormality, and no treatment was given. He was diagnosed with complex febrile convulsions. The boy started rhGH treatment (approximately 0.15 IU/kg/day, sc, qd) at 8 years and 4 months. Epilepsy occurred three times during the 6 months of rhGH treatment. Electroencephalography confirmed a definitive diagnosis of epilepsy. Then, he discontinued rhGH treatment at 8 years and 11 months and started taking levetiracetam (0.25 g, po, bid) for antiepileptic therapy. Epilepsy was well-controlled 4 months later. He continued rhGH treatment at 10 years and 3 months and has been on rhGH treatment until now, with no recurrence of epilepsy. He has been taking levetiracetam to date. Case 2, a 9-year and 1-month-old boy, was diagnosed with central precocious puberty, predicted short final height, and overweight. He started treatment with triptorelin (3.75 mg, im, q4w) and rhGH (approximately 0.15 IU/kg/day, sc, qd) at 9 years and 3 months. He tended to fall repeatedly when he was approximately 10 years old. Electroencephalography showed a few medium- to high-amplitude sharp waves and sporadic sharp slow waves in the left middle temporal region, sometimes involving the left posterior temporal region. He was diagnosed with epilepsy. Triptorelin discontinuance provided no symptom relief, which worsened further. Subsequently, he withdrew from rhGH treatment, and the symptoms occurred occasionally within a week and stopped after 15 days. The electroencephalogram returned to normal. No further seizures occurred during follow-up to date.

**Conclusion:**

During the use of rhGH in short-stature children with complex febrile convulsions or underlying lesions related to neurological impairment or those being treated with antiepileptic drugs, epilepsy may be induced.

## 1 Introduction

Recombinant human growth hormone (rhGH) is approved by the United States Federal Drug Administration for the treatment of growth hormone (GH) deficiency, chronic renal insufficiency, idiopathic short stature, Prader–Willi syndrome, short-stature homeobox-containing gene haploinsufficiency, Turner syndrome, Noonan syndrome, etc. (Graber et al., 2021). A cohort study in eight European countries showed no significant increase in the overall mortality for the low-risk group with isolated GH deficiency or idiopathic short stature after long-term rhGH treatment in childhood (Sävendahl et al., 2020). The increased overall mortality in moderate-risk and high-risk groups was associated with some potential or basal diagnosis, such as diseases of the circulatory and hematological systems. However, there was no correlation between the overall mortality and average or accumulated increasing doses of rhGH for all three groups, which confirmed the overall safety of rhGH treatment.

The rate of adverse events developed during rhGH treatment is approximately 6%–7% (Bell et al., 2010), including water–sodium retention, glucose metabolic disorder, slipped capital femoral epiphysis, scoliosis, hypothyroidism, hepatic dysfunction, tumor risk, and swelling at the injection site (Tao et al., 2015; Child et al., 2018; Bamba and Kanakatti Shankar, 2022). Few neurologically related adverse events were observed during rhGH treatment. Common neurological symptoms include headache and convulsions, which may be associated with hypertension or intracranial hypertension caused by water–sodium retention (Maghnie et al., 2022), yet there are few reports on epilepsy occurrence during rhGH treatment. In the full Kabi/Pfizer International Growth Database cohort for the safety and efficacy of rhGH therapy, treatment-related epilepsy accounted for 9.3% (4/43) of all-causality epilepsy (Maghnie et al., 2022). More studies on the safety monitoring of rhGH treatment are still needed to completely comprehend the potential adverse reactions and adverse events, providing data support for the specification of clinical rhGH treatment. For this reason, this study reports the case of two Chinese boys with short stature whose rhGH treatment was complicated with the occurrence of epilepsy and reviews the related literature aiming to alert clinicians to the possibility of induced epilepsy during the use of rhGH.

## 2 Case description

### 2.1 Case 1

A 7-year and 10-month-old Chinese boy, G1P1 with a gestation at 35 weeks, presented to our pediatric endocrinology clinic with the complaint of slow growth since childhood and congenital hypothyroidism. His birth weight was 2.23 kg, with unknown height and Apgar score. There was no history of asphyxiation resuscitation or birth injury. He was mainly breastfed after birth, and complementary foods were introduced on time. His growth and development retardation since childhood were manifested in obvious delays in movement and intellectual development, falling behind normal peers. The nutritional status was not good. He performed rehabilitation exercises for half a year. Vaccinations were not administered as scheduled. The height of his father and mother was 170 cm and 163 cm, respectively, and they were not consanguineous. His mother had a healthy pregnancy. There was no familial history of hereditary or contagious diseases.

The newborn heel blood screening showed that the thyroid-stimulating hormone level was a little high (data missed). He was diagnosed with congenital hypothyroidism and treated with levothyroxine sodium tablets for a long time. The parents self-stopped the medication when the boy was approximately 7 years old. From 6 to 7 years of age, the boy had suffered febrile convulsions more than 10 times. The main manifestations were daze, eye staring, action stopping, cold hands and feet, cyanosis of lips, inability to respond to calls, and no convulsions of limbs without obvious predisposing causes. Furthermore, the symptoms often take approximately 1–2 min to relieve. The electroencephalogram (EEG) and magnetic resonance imaging (MRI) showed no abnormality (detailed reports unavailable). He was diagnosed with complex febrile convulsions. No special treatment was given.

At the age of 8 years and 2 months, the boy underwent an examination related to short stature. His height was 109.1 cm (<P3), and the weight was 13.5 kg (P3∼P10) when evaluated (Li et al., 2009). His testicular volume was 2 mL, and both pubic and axillary hairs were in Tanner stage I. Thyroid function tests showed that T4 was 9.51 ug/dL, T3 was 122 ng/mL, free T3 was 3.71 ng/L, free T4 was 1.01 ng/dL, and thyroid-stimulating hormone was 4.88 uIU/mL, which was slightly high. The peak of GH during the GH stimulation test was 16.80 ng/mL, and IGF-1 was 120 ng/mL. There were no abnormalities in the levels of other biochemical indicators. The skeletal age was 8.4 years (TW2R method). The boy was definitely diagnosed with short stature, malnutrition, and congenital hypothyroidism. He was treated with rhGH (approximately 0.15 IU/kg/day, sc, qd) at 8 years and 4 months of age and continued peroral treatment with levothyroxine sodium tablets (35 ug/day, po, qd). Indicators such as thyroid function and glucose metabolism were monitored regularly during the treatment.

The boy presented with epilepsy three times during the 6 months of rhGH treatment (after 1, 2, and 7 months of rhGH treatment), at the age of 8 years and 5 months, 8 years and 6 months, and 8 years and 11 months, respectively. There was abdominal discomfort before the attack and dizziness, weakness, abdominal pain, and sweating after the attack, with no fever and no headache or projectile vomiting. The EEG showed the following abnormalities: sharp–slow waves and spike or slow waves dispersed or clumped in the forehead, frontal, parieto-occipital, and posterior temporal regions. The waves were significant in the left posterior head and occasionally generalized to all leads, and he was diagnosed with epilepsy. The complete EEG report is shown in Table 1. The rhGH injection was discontinued when the height and weight were 115.6 cm and 15 kg, respectively. Subsequently, he started levetiracetam (gradually increased to 0.25 g, po, bid; dosage by weight is approximately 29.4 mg/kg/day) for antiepileptic therapy. When coming for a follow-up visit at 9 years and 4 months of age, the boy had no further seizures. The EEG reviewed was generally normal, showing a pediatric borderline EEG with more fast waves in the frontal region during sleep, not excluding pharmacologic β-waves due to administration of antiepileptic drugs. The rhGH treatment was restarted at 10 years and 3 months of age. He continues to take levetiracetam (0.25 g, po, bid) to date. No seizures have occurred since then.

**TABLE 1:** Complete electroencephalogram after three occurrences of epilepsy during the use of rhGH in case 1.

**Background Activity**	When awake and quiet with eyes closed, 9–10 Hz medium-high amplitude rhythms were seen in the bilateral occipital areas, mixed with some 15–10 Hz β waves and scattered medium amplitude 5–7 Hz θ waves. Both sides are basically symmetrical, with poor amplitude modulation. Good cooperation with eyes open and closed. Eye-opening α-wave was suppressed.
**Hyperventilation**	Slow waves increased slightly.
**Flash Stimulation**	No abnormalities related to eye opening, closing, or IPS were observed.
**Sleep stages**	Sleep waves and sleep cycles were roughly normal.
**Abnormalities (Interictal)**	During awake and sleep periods, medium to high-amplitude 3–3.5 Hz sharp slow waves and spike (slow) waves were scattered or clustered in the frontal, forehead, vertex occipital, and posterior temporal areas, predominantly on the left side, occasionally generalizing to all leads, more prominent during the awake period.
**Abnormalities (Ictal)**	No special clinical events were monitored.
**Impression**	Scattered or clustered sharp slow waves and spike (slow) waves in the frontal, forehead, vertex occipital, and posterior temporal areas, predominantly in the left posterior head, occasionally generalizing to all leads.

### 2.2 Case 2

A 9-year and 1-month-old Chinese boy visited the pediatric endocrinology clinic of our hospital due to concerns about early puberty and obesity. The boy, G3P1, was delivered at full term with a birth weight of 3.2 kg and a height of 50 cm. The Apgar score was unknown. There was a history of neonatal pneumothorax but no birth trauma history. His parents’ height was 163 cm and 158 cm, and they were not consanguineous. His grandmother’s height was 143 cm. His mother’s first menstrual period occurred at approximately 14 years of age. There was no family history of hereditary diseases.

A physical examination showed that the height and weight of the boy were 137 cm and 37 kg, respectively, and the BMI was 19.7 kg/m^2^. The testicular volume was 5 mL, and the length of the penis was 6.5 cm. His bone age was 11.7 years (TW2R method). The peak luteinizing hormone after the gonadotrophin-releasing hormone analog (GnRHa) stimulation test was 9.67 IU/L. The blood glucose, insulin, and other metabolic indicators were normal. The boy was diagnosed with central precocious puberty (CPP), predicted short final height, and overweight. At the age of 9 years and 3 months, the child was treated with triptorelin (a type of GnRHa, 3.75 mg per time, intramuscular injection, q4w) and rhGH (approximately 0.15 IU/kg/day, sc, qd). The child was followed up regularly during treatment.

At the age of 10 years, the boy started to walk shakily and fell repeatedly. The seizure occurred 2–3 times per day, was controllable, and lasted for a few seconds. There were no obvious jerking movements of the limbs or other discomfort, such as headache and projectile vomiting. After the symptoms lasted for 3 months, the boy discontinued triptorelin. Subsequently, the frequency of the seizure decreased to 1–2 times per day, but the symptoms worsened. He knocked out half an incisor after a severe fall.

At 10 years and 6 months, he presented to the pediatric neurology department. The 24-h EEG showed symmetric low-voltage α-waves (9–10 hz) in both hemispheres, with multiple sharp and sharp–slow waves noted in the left temporo-parietal region. EEG + topographic image impression displayed abnormal pediatric EEG, with a small number of medium- and high-amplitude sharp waves in the left middle temporal region, sporadic sharp and slow waves, and sometimes spreading to the left posterior temporal region. When he was 10 years and 6 months old, he stopped rhGH treatment. After stopping the medication, seizures occurred occasionally within 1 week and stopped after 15 days. One month later, the EEG returned to normal. No further seizures happened since the follow-up.

## 3 Discussion

In this report, we presented detailed information of two Chinese boys who developed epilepsy during rhGH treatment or rhGH combined with GnRHa treatment. According to the World Health Organization–Uppsala Monitoring Center system for standardized case causality assessment (the use of the WHO), we evaluated the causal relationship between epilepsy and rhGH or GnRHa treatment. In both cases 1 and 2, the causal relationship between epilepsy and rhGH treatment was considered to be possible. In case 2, the causal relationship between epilepsy and GnRHa treatment was considered unlikely.

Epilepsy is a clinical syndrome resulting from a highly synchronized abnormal discharge of brain neurons due to various reasons (Falco-Walter, 2020). Secondary epilepsy can be attributed to many reasons, such as structural, genetic, infectious, metabolic, drug-induced, and immunologic causes. A large number of known medicines have been associated with seizures, including antipsychotics (clozapine), fluoroquinolones (ofloxacin), antitubercular agents (isoniazid), tricyclic antidepressants (imipramine), glucocorticoids (dexamethasone), nonsteroidal anti-inflammatory drugs, and chemotherapeutic drugs (platinum and cyclosporine) (Williams and Park, 2015; Walton et al., 1997; Larson et al., 2021). However, reports on the association of epilepsy with endocrine-related drugs such as GnRHa and rhGH are rare.

To date, studies on whether GnRHa can induce or improve epilepsy are scarce and divergent. The temporal lobe limbic structures, which are anatomically often associated with epilepsy, are highly connected to the hypothalamus. Both gonadotropins and gonadotrophin-releasing hormone (GnRH) can modulate the function of hippocampal neurons and other extra-hypothalamic regions to influence seizures (Cutia and Christian-Hinman, 2023). Furthermore, epilepsy can affect the release of gonad-related hormones, possibly leading to anovulatory menstruation, decreased fertility, or early menopause in women with epilepsy, suggesting a certain interrelationship between epilepsy and the hypothalamic–pituitary–gonadal axis (Markoula et al., 2020).

Some studies suggest that GnRHa treatment may be a trigger for epilepsy. It was reported that a 13-year-old girl was treated with leuprolide acetate (LA) (3.75 mg) for menstruation after chemotherapy and radiotherapy for medulloblastoma in the fourth ventricle (Akaboshi and Takeshita, 2000). After the third LA injection caused an atypical absence seizure, LA was discontinued, no antiepileptic drugs were given, and then the epilepsy ceased. Similar seizures recurred 2 years and 6 months later. According to the endocrinology examination results and LA pharmacokinetic report data, it was speculated that the seizure might be related to LA and the presence of brain damage, so it is recommended that caution should be taken in the treatment of LA or other types of GnRHa for children with diffuse brain injury. Another girl with hemiplegia caused by neonatal cerebral infarction was reported to develop complex partial seizures at 4 years of age and started treatment with sodium valproate (Minagawa and Sueoka, 1999). Seizures recurred at the age of 7 years and were suppressed with clonazepam. When LA treatment was initiated for CPP at 8 years and 4 months of age, the first seizure occurred 72 days after treatment. Subsequently, it occurred frequently, and the adjustment of antiepileptic drugs did not work, but the occurrence of seizures sharply decreased after discontinuing LA administration. The decrease in the serum progesterone concentration caused by LA might lead to an increase in epilepsy occurrences, suggesting that attention should be paid to the possibility of inducing epilepsy during LA treatment. A 7-year and 2-month-old girl started antiepileptic treatment for epileptic myoclonic atonic seizures and GnRHa for CPP (Proietti et al., 2024). Status epilepticus first appeared 1 week after the first GnRHa injection. GnRHa therapy was suspended, and it was successfully treated with antiepileptic therapy. Therefore, GnRHa may be associated with the occurrence of status epilepticus. During the use of GnRHa in patients with diffuse brain injury, neonatal cerebral infarction, or antiepileptic treatment, epilepsy may be induced and even aggravated.

It has also been suggested that GnRHa plays a role in improving epilepsy symptoms. Two children (one girl and one boy) with hypothalamic hamartoma, precocious puberty, and epilepsy were reported to have their seizures relieved after receiving GnRHa treatment (Zaatreh et al., 2000). Similarly, 10 premenopausal women with catamenial epilepsy were treated with antiepileptic drugs with triptorelin (3.75 mg, q4w), with 3 of them having no further seizures, 4 having reduced seizure frequency, and 1 having shorter seizure duration (Bauer et al., 1992). This suggests that triptorelin may be helpful for patients with refractory catamenial epilepsy. The subjects in the above studies are predominantly women, but few relevant studies are focused on men. In our study, case 2 is a boy with no underlying disease, and the frequency of epilepsy occurrence decreased slightly after stopping triptorelin, but the symptoms did not alleviate, rather they aggravated. The symptoms were relieved entirely after discontinuing rhGH. Therefore, the epilepsy in the boy may not be related to triptorelin. Compared with female mice, temporal-lobe epilepsy has less effect on the activity and excitability of GnRH neurons in male mice, indicating sexual dimorphism in the effect of temporal-lobe epilepsy on GnRH (Li et al., 2018). It is indirectly implicated that there may be little correlation between epilepsy and GnRHa treatment in men, which is essential for understanding the relationship between seizures and GnRHa use in case 2 of this study and girls in the literature. From this point of view, LA should be alert to the possibility of epilepsy in girls with underlying brain disease. Triptorelin may be effective in menopausal women with catamenial epilepsy. In girls with underlying disease, concomitant CPP requiring GnRHa treatment, controversy exists as to whether the treatment induces or ameliorates epilepsy. In the case of the two boys reported by our team, it is suggested that GnRHa may not be strongly associated with seizures, yet this has to be further analyzed in the accumulated clinical cases.

Likewise, there is less research on rhGH-associated epilepsy. The latest multicenter longitudinal observational study analyzing the long-term safety of rhGH treatment showed that the most common adverse drug reactions were nervous system disorders with an incidence of 30.5% (236/774), among which headache, with an incidence of 26.5% (205/774), was the most frequent (Sävendahl et al., 2021). Furthermore, nervous system disorders were also the most common events for serious adverse events with an incidence of 18.8% (123/656), and epilepsy accounted for 6.7% (44/656) of serious adverse events. For serious adverse drug reactions, the incidence of nervous system disorders was 18.9% (36/190), including headaches, with an incidence of 6.3% (12/190). The potential relationship between rhGH treatment and headaches could not be analyzed due to the limitations of low symptom specificity and heterogeneity of concomitant diagnoses, not to mention epilepsy. In our study, case 1 had a history of congenital hypothyroidism; his growth and development retardation since childhood was manifested in obvious delays in movement and intellectual development, falling behind normal peers. This indicated that he had neurological impairment. At the age of 6–7 years, he was diagnosed with complex febrile convulsions. During the therapy for short stature, he developed epilepsy after 1, 2, and 7 months of rhGH treatment. Case 2, without any underlying disease, experienced epilepsy after 9 months of rhGH, essentially ruling out the possibility of epilepsy induced by triptorelin (symptoms of seizure worsened after triptorelin was discontinued). As can be seen, epilepsy may be one of the neurological adverse effects that cannot be ignored during rhGH treatment, so clinicians need to be cautious about the possibility of epilepsy in children during the rhGH treatment. Moreover, epilepsy may be more easily induced when the patient has basic diseases such as complex febrile convulsions or underlying lesions related to neurological impairment. More related clinical cases should be studied further.

To date, the potential association and mechanism between epilepsy and rhGH are still uncertain. First, it has been shown that epilepsy during rhGH treatment may occur through the induction of GH signaling transportation. The rhGH injection into the hippocampus of male mice could lead to a significant enhancement in seizures, affect the process of behavioral epileptogenesis, and increase the spike number, after discharge durations, and threshold, which could demonstrate that rhGH is one of the essential causes in the development of epilepsy (Kato et al., 2009). Second, some studies suggested that GH-specific binding sites in some regions of the central nervous system, which controlled sleep, cognition, emotion, and neuroprotection, were all being affected by GH secretion (Butler et al., 2019). GH is expressed in the brain and can cross the blood–brain barrier, and GH receptors are widely expressed in several regions of the rodent and human brain including the hippocampus region (Arámburo et al., 2014). Finally, GH plays a vital role in regulating different neurotransmitter concentrations of cerebrospinal fluid. The rhGH treatment may increase aspartate levels by 30%, a major mediator of excitatory synaptic transmission, which could be involved in the pathophysiologic alterations associated with epileptogenesis (Devesa et al., 2010). In addition, GH was reported to have a huge effect on the hippocampal synaptic transmission of rats by modulating N-methyl-D-aspartate receptor-mediated excitatory postsynaptic potentials (Mahmoud and Grover, 2006). The primary manifestation was that GH enhanced N-methyl-D-aspartate receptor-mediated excitatory postsynaptic potentials in the CA1 region of the hippocampus, with no alteration in the γ-aminobutyric acid receptor-mediated inhibitory synaptic transmission.

In summary, this study demonstrates that epilepsy occurred during rhGH treatment in two boys, indicating that rhGH may have specific effects and functions in the central nervous system, which suggests that the possibility of neurological diseases such as epilepsy should not be overlooked in children undergoing rhGH therapy. Therefore, pediatric endocrinologists should inquire about any underlying brain diseases, convulsions, or history of epilepsy in the child before administering rhGH. They should communicate with the parents that epilepsy may be induced during rhGH treatment, especially in short-stature children with complex febrile convulsions and underlying lesions related to neurological impairment or those being treated with antiepileptic drugs. Throughout the process of using rhGH to improve the final height of the children, it is crucial to closely monitor for any neurological symptoms related to epilepsy. If similar symptoms occur, the medication should be terminated immediately and symptomatic treatment provided. Decisions on whether rhGH treatment can be continued later should be made after a comprehensive assessment of the child’s condition by pediatric endocrinologists and pediatric neurologists.

## Data Availability

The original contributions presented in the study are included in the article/Supplementary Material; further inquiries can be directed to the corresponding authors.
